# Sources of Dietary Fiber and the Association of Fiber Intake with Childhood Obesity Risk (in 2–18 Year Olds) and Diabetes Risk of Adolescents 12–18 Year Olds: NHANES 2003–2006

**DOI:** 10.1155/2012/736258

**Published:** 2012-08-23

**Authors:** Mary Brauchla, WenYen Juan, Jon Story, Sibylle Kranz

**Affiliations:** ^1^Department of Nutrition Science, Purdue University, 204 Stone Hall, 700 W. State Street, West Lafayette, IN 47907, USA; ^2^Office of Nutrition, Labeling and Dietary Supplement, Center for Food Safety and Applied Nutrition, U.S. Food and Drug Administration, Department of Health and Human Services, College Park, MN 20740, USA

## Abstract

Increased fiber intake has been linked with lower risk of overweight and obesity in adults, but data are sparse for children. To address this issue, NHANES 2003–2006 data was used to evaluate (1) the food sources of fiber in children, (2) the dietary fiber density levels and risk of being classified as overweight/obese, and (3) the association between fiber intake level and impaired glucose metabolism in children. Analyses were restricted to the subsample of children with biological plausible diet reports (*N* = 4,667) and stratified by 2–11 year olds (*n* = 2072) and 12–18 year olds (*n* = 2595). Results showed that the food sources are predominantly foods that are low in dietary fiber, but are consumed at high levels. In 2–18 year old plausible reporters, the risk for overweight/obesity decreased by 17% from children in the medium tertile of fiber density intake compared to the lowest tertile (OR = 0.83, *P* value = 0.043) and by 21% between the highest compared to the lowest tertile (OR = 0.79, *P* value = 0.031). There was a protective effect of being in the medium tertile of dietary fiber density (OR = 0.68, *P* value <0.001) on impaired glucose metabolism. These results indicate a beneficial effect of higher fiber density in children's diets.

## 1. Introduction

Considerable evidence supports that increasing consumption of dietary fiber is associated with lower risk of obesity in adults [1]. Based on these data, the Dietary Reference Intakes (DRIs) for Americans two years and older is to consume 14 grams (g) of total fiber per 1000 kilocalories (kcal) of total energy intake. In younger children, a two-year follow-up study conducted in Latino children 7–11 years old showed that an increase of 3 g of dietary fiber/1000 kcals was associated with a 4% reduction of visceral body fat while a fiber decrease of the same amount was associated with a 21% increase of visceral body fat [2]. In addition, overweight Latino children who consumed 5.2 g of soluble fiber were more likely to have none of the features of metabolic syndrome while those who consumed 4.1 g were more likely to have 3+ features [3]. Others failed to establish a clear relationship between fiber intake levels and body weight, possibly due to the length of the study or the different types of fiber or food sources. 

Obesity in childhood often results in insulin resistance, which disrupts glucose metabolism and can eventually result in diabetes [4], but data on glucose metabolism and fiber intake are sparse for children. Results of a meta-analysis of studies from 1980 to 2010 suggest that increased fiber intake is associated with decreased blood glucose and glycosylated hemoglobin (HbA1c) in adults [5]. In addition, a review on psyllium fiber showed that increased psyllium consumption resulted in improved glucose homeostasis, with postprandial glucose values decreasing by 12.2% to 20.2% in children with type 2 diabetes [6]. However, research results are inconsistent, which may be due to the dissimilar effects of soluble and insoluble fiber. Current research suggests that cereal fiber and whole-grain intake is associated with decreased risk of diabetes, but no effect was found with increased intake of soluble fiber from fruits and vegetables [7, 8]. These data are supported by research indicating that consumption of cereal fiber and whole grains increase insulin sensitivity in overweight and obese adults [9, 10]. 

To examine the issue, the objectives of this study were to use the data from 2–18 year old American children with plausible intake reports in a nationally representative data set and to (1) determine dietary fiber sources in children's diets, (2) investigate the association between dietary fiber intake level and body weight status in 2–11 and 12–18 year old children, and (3) investigate the association between dietary fiber intake level and impaired glucose metabolism in teenagers 12–18 years old, who provided fasting blood samples. 

## 2. Methods

### 2.1. Data Used

We used socioeconomic, dietary, and medical examination data from the combined survey years of 2003-2004 and 2005-2006 of the National Health and Nutrition Examination Survey (NHANES (available at http://www.cdc.gov/nchs/nhanes.htm)). During the survey, an adult was chosen for the household interview and reported sociodemographic information, such as gender, age, race, ethnicity, and household income. For this study two mutually exclusive age groups, 2–11 and 12–18 year olds, were created to account for the different eating patterns in these two groups.

According to the interview responder's categorization, race and ethnicity were reported as American Indian or Alaskan Native, Asian, black or African American, Native Hawaiian or Pacific Islander, White, or other non-Hispanic, Mexican American, other Hispanic. These variables were recoded to reflect the cultural eating differences in Hispanic/other (Mexican Americans, other Hispanic, other/multiethnic), Non-Hispanic White, and Non-Hispanic black children. Household income was used to differentiate households by the income-eligibility cut-points for USDA food assistance programs; high income was defined as ≥3.5 of the Poverty Income Ratio (PIR), medium income defined as 1.86–3.4 PIR, and low income defined as ≤1.85 PIR. The latter group are income eligible for participation in the USDA food assistance program [11]. The PIR is used routinely to express the available income of households, accounting for the number of individuals living in the household. The distribution of the sample population is reflected in Table 1.

### 2.2. Dietary Data

Two 24-hour dietary recalls of food consumption data are available for both 2003-2004 and 2005-2006 survey years. Detailed information on survey design and data collection can be found elsewhere [12]. To accommodate the increasing food intake with older age, the sample was divided into two groups: 2–11 year olds and 12–18 year olds for all analysis.

#### 2.2.1. Person-Level Intake Data

For this study, only two-day total energy and dietary fiber data were calculated for each child in the data set. To provide a direct comparison to the DRI recommendation for total fiber intake, dietary fiber density in children's diets was calculated (average grams of fiber per 1000 kcal total energy consumed). This dietary fiber density variable was used to create three levels of fiber consumers in tertiles of fiber density.

#### 2.2.2. Plausibility of Reported Diets

Due to under- or overreporting of dietary data in certain groups [13], plausibility of the diet data was determined. Biological plausibility of the dietary energy intake records was ascertained using the method described by Huang et al. [14]. In short, children's reported energy intake was compared to calculated, plausible age-and-gender specific energy expenditure data. Due to the bias introduced by unreliable intake reporting, all analyses of this study were based only on children who had plausible intake data.

#### 2.2.3. Food-Level Intake Data

To explore the food sources of dietary fiber, food-level analysis was conducted. One day of 24-hour recall data was randomly chosen to obtain the most accurate amounts of fiber contribution for each food consumed. This method is routinely used to address the issue of daily variation of dietary intakes. For instance, a child might eat one egg on day one but no eggs on day two of the study. However, when examining food sources, one should not conclude that the child eats half of an egg every day. Randomly choosing one day of the intake data is a feasible method for this type of analysis. The top 20 food codes in children ages 2–11 and 12–18 are presented in Table 2.

#### 2.2.4. Body Weight Status and Risk for Diabetes

Anthropometric data were obtained when NHANES participants visited the Mobile Examination Center (MEC). Using standard procedures, height and weight were measured, and children were classified as being underweight, normal weight, overweight, or obese using the standards published by the CDC (body-mass-index (BMI) weight-for–age growth charts [15] in that overweight included children whose BMI-for-age percentile was between the 85th and 94th percentile and obese if it was larger or equal to the 95th percentile. For the purpose of this study, children who were underweight (less than the 5th percentile, 3% of the sample) were combined with those who have healthy body weight (6th−84th percentile, 58% of the sample). Venous blood samples were drawn only in children at least 12 years old using standard procedures, and fasting blood glucose levels were ascertained using an enzyme hexokinase (HK) method. A fasting glucose level of at least 126 milligrams per deciliter (mg/dL) was used as the cut-point to establish impaired glucose metabolism. Full details on NHANES methodology to estimate impaired glucose metabolism can be found at http://www.cdc.gov/nchs/nhanes.htm. 

### 2.3. Examination of Diet-Disease Associations, Data Analysis

The statistical software STATA 11 (Stata Corp, version 11) was used for all data analysis. Survey routine procedures were used to account for NHANES's complex survey design and the sampling weights. The 4-year medical sampling weights were used to generate descriptive statistics and for the logistic analysis. Food-level analysis was based on one day of intake, and the calculation of weighted total intake of dietary fiber was performed using the one-day dietary intake weight (WTDRD1). The relationship between the dietary fiber level in children's diets and their risk for being overweight or obese was estimated using multiple, logistic regression models. The independent variable was expressed in tertile of fiber density (referent, lowest tertile) while the main dependent variable was coded as a dichotomous variable (overweight/obese (=1) or not (=0)). Analyses were conducted by age group, controlling for age (as continuous variable), gender, ethnicity, and income as well as ethnicity and income interactions. Results were reported as odds ratios (ORs) with 95% confidence intervals. Similarly, the association between dietary fiber intake level and the risk for impaired glucose metabolism was examined using logistic regression models where the independent variable was expressed in tertile of fiber density (referent, lowest tertile) while the main dependent variable was coded as a dichotomous variable (impaired glucose metabolism (=1) or not (=0)). Analyses were also controlled for gender, ethnicity, and income as well as ethnicity and income interactions. Due to the lack of data using fasting blood samples in children younger than 12 years old, the analysis was restricted to children 12–18 years old, who provided fasting blood samples. Significance level for all analysis was set at *P* < 0.05. 

## 3. Results

The population characteristics are shown in Table 1. The total population included *N* = 6556 children, representing 109,068,577 children ages 2–18 years old in the US population. Within the total population, *N* = 4755 children, approximately 80% of the sample, had provided biological plausible intake reports and were included in the analysis. There were significant differences in the ethnic background of the plausible reporters versus the nonplausible reporters. In that more non-Hispanic black and Mexican American than non-Hispanic white children provided plausible reports, likewise, more low-income children than medium- or high-income children were plausible reporters.

In both age groups, more children who were plausible reporters had healthy weight (46% and 54% of 2–11 and 12–18 year old, resp.). Overweight children who were plausible intake reporters comprised 9% of the 2–11 year olds and 13% of the 12–18 year olds, while obese plausible intake reporters comprised 19% of the 2–11 year olds and 14% of the 12–18 year olds. 

Examination of the food sources of fiber revealed that the foods providing the highest proportions of fiber to the plausible diets of children were not high-fiber foods, such as French fries and pizza or white bread/rolls (Table 2). The main sources of fiber in the 2–11 year old children with healthy weight compared to the overweight/obese showed several differences; healthy weight children consumed more high-fiber foods such as peanut butter or popcorn compared to the overweight/obese. The food that contributed the highest amount of dietary fiber in children ages 2–11 and 12–18 years old was the “meatless bean and cheese burrito,” which was consumed in amounts that contributed the highest amount of fiber: up to 30.8 g of dietary fiber in the 2–11 year olds and up to 48.3 g in 12–18 year olds. Other foods consumed in amounts that rendered them high contributors of dietary fiber included baked white potatoes, beans and franks, canned pasta with tomato sauce and meat/meatballs, refried beans, and chili con carne. 

Average total dietary fiber density was 6.4 g/1000 kcals in plausible reporters age 2–18. Dietary fiber density intake in 2–11 year old children was 6.68 g/1000 kcals, significantly higher than the dietary fiber density intake for 12–18 year old children of 6.15 g/1000 kcals (*P* < 0.001). Tertiles of average total dietary fiber density for 2–18 year old plausible reporters were 4.4, 6.1 and 8.8 g fiber/1000 kcals for the lowest, medium and highest tertiles. In 2–11 year old plausible reporters, the fiber density intake tertiles were 4.5, 6.2, and 8.8 g/1000 kcal of the lowest, medium, and highest tertile, respectively. In 12–18 year olds, tertiles of dietary fiber intake density were 4.3, 6.1, and 8.9 g/1000 kcal for the lowest, medium, and highest tertiles. Thus, on average dietary fiber intake levels were less than half of the DRI.

Odds ratios for disease risk factors of BMI for children 2–18 years old are shown in Table 3. In 2–18 year old plausible reporters, the risk for overweight/obesity decreased significantly by 17% from children in the medium tertile of fiber density compared to those in the lowest tertile (OR = 0.83, *P* value = 0.043) and by 21% between those in the highest compared to the lowest tertile (OR = 0.79, *P* value = 0.031). There was a trend of decreasing risk for overweight/obesity with increasing fiber density among respondents 2–11 years old, but this trend was not significant. In 12–18 year olds with plausible diet records, the risk for overweight/obesity decreased by 25% from children in the highest fiber density intake tertile compared to the lowest tertile (OR = 0.75, *P* = 0.043). Analysis of further subgrouping of the population yielded mixed results. Due to the small sample size of plausible reporters in the age groups, logistic models using those strata were not examined. 

Of the 2,709 teenagers 12–18 years old who provided fasting blood glucose samples, 2661 were plausible reporters, and 1508 were plausible reporters with impaired blood glucose. A large protective effect of being in the medium tertile of dietary fiber density was found (OR = 0.68, *P* value <0.001), but this trend was not significant from children in the highest tertile compared to the lowest (OR = 0.75, *P* = 0.070) (Table 3).

## 4. Discussion

The NHANES data are collected continuously to address the government's mission to ensure the health and wellbeing of the American people via nutrition monitoring. This study provides information on the sources of fiber and the association between total dietary fiber intake and the odds of selected chronic diseases in children. To pursue this goal, NHANES data of the subsample with biological plausible intake reports were examined and stratified by age group. Furthermore, data were analyzed on the population and on the person level to explore the foods contributing most fiber to children's diets.

Rates of overweight and obesity in this sample are consistent with previous research [16]. The results shown here indicate a lower risk for childhood obesity with increasing dietary fiber intake. A recent review of the literature suggested a number of mechanisms by which dietary fiber intake might help downregulate body weight in adults [17].

Data on fiber intake and body weight in the pediatric population are not consistent. Lack of dietary fiber in children's diets was associated with higher body fatness in a sample of British children [18] and 15 g of supplemental fiber in addition to a calorie-restricted diet resulted in 2 kg more weight loss [19]. Others found no association between dietary fiber and adiposity [20]. In a sample of German children, higher fiber density was associated with increased risk for overweight/obesity [21]. One longitudinal study with two year follow-up showed that 7–11 year old Latinas who consumed higher levels of soluble fiber had a small but significant reduction of visceral body fat; on the other hand, lower fiber intake was associated with a 10% increase of visceral body fat [2]. Increasing dietary fiber intake by the equivalent of 1/2 cup of beans per day to children's diets for 16 weeks resulted in decreased visceral adipose tissue by 10% in overweight Latino adolescents [22]. Conflicting results may be due to the different time periods, populations, and the amount and type of fiber assessed in these studies. 

One possible venue for the beneficial effect of fiber on weight status could be the increase in satiety [23]. Postprandial glucose levels and increased insulin sensitivity are associated with the increased viscosity of soluble fiber intake which has been associated with delayed gastric emptying, altering of gastrointestinal myoelectrical activity, decreased glucose diffusion through the water layer, and decreased accessibility of substrates to *α*-amylase—thus increasing satiety [23, 24]. Insoluble fiber, on the other hand, does not absorb water but increases insulin sensitivity; a clear mechanism or pathway for this phenomenon has not been shown to date [25].

The majority of research on fiber intake and glucose metabolism focuses on adults, and data in children are scarce. Increased fiber density intake is inversely associated with impaired glucose tolerance in Finnish adults [26]. Others found that consumption of 10 g of *β* glucan by obese women resulted in significantly decreased glucose response after 30 minutes as well as a delayed glucose response [27]. Furthermore, increasing cereal fiber by 31.2 g/day for 3 days resulted in improved insulin sensitivity in overweight and obese women [28]. Diabetic adults who consumed a diet with 50 g of fiber for 6 weeks had significantly reduced their preprandial plasma glucose and area under the curve for 24-hour plasma glucose compared to those who consumed a diet with 25 g of fiber that was identical in macronutrient and energy content [29]. A review by Moreno et al. found that psyllium supplementation resulted in a 12%–20% reduction in postprandial glucose levels in children and adolescents with type 2 diabetes [6]. Psyllium fiber in particular has been identified as a method to decrease glucose levels in diabetic adults and has even been suggested as an additional treatment to type 2 diabetics [30].

The mechanism behind this phenomenon has not been established, but Weickert et al. have proposed a theory involving the mTOR pathway [9]. This theory is based on results of their recent study suggesting that obese adults consuming a diet high in protein have reduced insulin sensitivity and higher expression of protein ribosomal subunit serine kinase 6-1 (S6K1) while adults consuming a high-protein and high-cereal-fiber diet had S6K1 levels similar to baseline values. These data expand on previous research indicating that the inhibition of glucose uptake is associated with phosphorylation of downstream targets of S6K1 [31]. Weickert et al. hypothesize that the stable S6K1 expression in adults consuming the high protein and cereal fiber is due to fiber's interference in the digestion and/or absorption of the protein. This theory may provide further insight to the effects of fiber on insulin sensitivity, and additional research should be initiated.

 Our findings support previous research and suggest that fiber intake is associated with improved glucose metabolism. However, due to the relatively small number of individuals who were plausible reporters and had higher fiber intakes and the resulting intake distribution curves that were drastically skewed to the left, we were not able to show a consistent trend in improved health status with increasing level of dietary fiber intake. 

A number of limitations affected this study. The most critical issue was that only dietary but not functional fiber intake can be estimated when using the NHANES data. Furthermore, only total dietary fiber but not soluble or insoluble fiber is included in the data set; thus, the specific effects of these two types of fibers could not be discerned. One major strength of this study was the use of a nationally representative dataset to explore diet-disease relationships. Epidemiologic and population-based studies are of tremendous value in the examination of diet-disease associations. However, the nature of such data precludes any indication of causality, and data are limited to the variables provided, making it impossible for researchers to examine other factor of interest. In addition, the accuracy of self-reported dietary intake data inherent to those studies is often a limiting factor. Since it is not possible to objectively and directly measure dietary intake in such large samples, individuals can under- or overreport all or selected foods, introducing reporting bias which results in skewed data [32]. Thus, studies investigating diet-obesity relationships should include the examination of biological plausibility in the intake data, as was performed here. 

## 5. Conclusions

Although our results show a beneficial association between dietary fiber intake and lower risk for overweight and obesity, longitudinal studies clearly establishing a causal relationship are needed. Most children in this study underconsumed dietary fiber by more than 60% and even children with diets in the highest fiber density tertile failed to meet the intake recommendation, with younger children having a higher fiber density intake than older children. We showed that high-fiber diets were the result of large food intake, not the consumption of fiber dense foods. The potentially beneficial effect of the fiber on children's health is diluted by excessive energy intake; therefore, children should be encouraged to consume fiber-rich foods, such as fruits, vegetables and whole grains. More research is needed to identify interventions to increase the fiber density of children's diets with the goal of lowering childhood obesity.

## Figures and Tables

**Table 1:** Population characteristics of children 2–11 and 12–18 years old with plausible diet records, NHANES 2003–2006 (in percent).

	Plausible energy reporters 2–11 years old Weighted percent (*n* = 2072)	Plausible energy reporters 12–18 years old Weighted percent (*n* = 2595)
Gender		
Males	53.0	52.4
Females	47.0	47.6
Ethnicity		
Non-Hispanic White	60.7	65.2
Non-Hispanic Blacks	13.6	13.8
Hispanic/other race	25.8	20.9
Income		
PIR^1^ < 185%	40.6	34.0
PIR^1^ ≥ 185% and <350%	29.0	28.4
PIR^1^ ≥ 350% and <500%	30.5	37.6

^1^PIR: poverty income ratio, values >5.0 were recorded as 5.0 in the NHANES.

**Table 2:** Foods contributing the highest proportion of dietary fiber^1^ for plausible energy reporters aged 2–11 and 12–18 by weight status.

Food ranking	Healthy weight	Overweight/obese
Ages 2–11		

1	Burrito with beans and cheese, meatless	Burrito with beans and cheese, meatless
2	Banana, raw	Apple, raw
3	Apple, raw	White potato, French fries
4	White potato, French fries	Frosted Mini-Wheats cereal (all flavors)
5	Spaghetti with tomato sauce and meat sauce	Bread, white
6	Bread, white	Salty snacks, corn or cornmeal, tortilla chips
7	Pizza, cheese, thin crust	Orange, raw
8	Pizza with meat, thin crust	Banana, raw
9	Orange, raw	Spaghetti with tomato sauce and meat sauce
10	Pizza with meat, thick crust	Waffle, plain
11	Popcorn, popped in oil, buttered	White potato, chips
12	Peanut butter	Tortilla, corn
13	Spaghetti with tomato sauce, meatless	Burrito with beef and beans (include burrito)
14	Macaroni or noodles with cheese	Baked beans, vegetarian
15	Roll, white, soft	Milk, chocolate, red fat, 2%
16	Salty snacks, corn or cornmeal, tortilla chips	Pizza with meat, thick crust
17	Carrots, raw	Refried beans
18	Tortilla, corn	Pizza, cheese, thick crust (including English muffin)
19	Milk, chocolate, red fat, 2%	Pizza with meat, thin crust
20	White potato, chips	Carrots, raw

Ages 12–18		

1	White potato, French fries	Pizza with meat, thick crust
2	Burrito with beans and cheese, meatless	White potato, French fries
3	Pizza with meat, thick crust	Salty snacks, corn or cornmeal, tortilla chips
4	Roll, white, soft	Apple, raw
5	Apple, raw	Bread, white
6	Spaghetti with tomato sauce and meat sauce	Pizza with meat, thin crust
7	White potato, chips	Spaghetti with tomato sauce, meatless
8	Salty snacks, corn or cornmeal, tortilla chips	Banana, raw
9	Banana, raw	Tortilla, corn
10	Raisin Bran, Kellogg's	Carrots, cooked, fat not added
11	Pizza with meat, thin crust	Burrito with beef and beans (include burrito)
12	Peanut butter	Frosted Mini-Wheats cereal (all flavors)
13	Bread, white	White potato, chips
14	Bread, wheat or cracked wheat	Spaghetti with tomato sauce and meat sauce
15	Chicken patty/fillet/tenders, breaded, cooked	Sausage, potato and vegetables with gravy
16	White potato, French fries	White potato, French fries
17	Baked beans, vegetarian	Roll, white, soft
18	Tortilla, flour (wheat)	Bread, wheat or cracked wheat
19	Pasta with tomato sauce and meat/meatballs, canned	Bagel
20	Popcorn, popped in oil, buttered	Sunflower seeds, hulled, roasted, salted

^1^ Proportion of dietary fiber is calculated as grams of total dietary fiber from food item/total gram of dietary fiber consumed.

**Table 3:** Odds ratio (OR) for disease risk factors of body mass index (BMI) for children aged 2–18 and fasting blood glucose for children aged 12–18 by 2-day average intake tertiles of total dietary fiber density for plausible energy reporters controlling for age, gender, ethnicity and income.

	Medium tertile			Highest tertile		
	OR	95% CI	*P* > chisq	OR	95% CI	*P* > chisq
Disease risk factor						
Overweight/obese by body mass index for 2–18 year old children^1^	0.83	(0.69, 0.99)	0.043	0.79	(0.63, 0.98)	0.031
Overweight/obese by body mass index for 2–11 year old children^1^	0.87	(0.69, 1.10)	0.224	0.87	(0.64, 1.18)	0.365
Overweight/obese by body mass index for 12–18 year old children^1^	0.84	(0.61, 1.16)	0.274	0.75	(0.56, 1.00)	0.043
High fasting glucose for 12–18 year old children^2^	0.68	(0.54, 0.85)	<0.001	0.75	(0.55, 1.02)	0.070

^1^For age 2–18, body mass index is above 85th percentile.

^2^Fasting blood glucose level is more than 126 mg/dL.

## References

[1] Anderson JW, Baird P, Davis RH (2009). Health benefits of dietary fiber. *Nutrition Reviews*.

[2] Davis JN, Alexander KE, Ventura EE, Toledo-Corral CM, Goran MI (2009). Inverse relation between dietary fiber intake and visceral adiposity in overweight Latino youth. *American Journal of Clinical Nutrition*.

[3] Ventura EE, Davis JN, Alexander KE (2008). Dietary intake and the metabolic syndrome in overweight latino children. *Journal of the American Dietetic Association*.

[4] Weiss R, Caprio S (2006). Altered glucose metabolism in obese youth. *Pediatric Endocrinology Reviews*.

[5] Post RE, Mainous AG, 3rd, King DE, Simpson KN (2012). Dietary fiber for the treatment of type 2 diabetes mellitus: a meta-analysis. *Journal of the American Board of Family Medicine*.

[6] Moreno LA, Tresaco B, Bueno G (2003). Psyllium fibre and the metabolic control of obese children and adolescents. *Journal of Physiology and Biochemistry*.

[7] Schulze MB, Schulz M, Heidemann C, Schienkiewitz A, Hoffmann K, Boeing H (2007). Fiber and magnesium intake and incidence of type 2 diabetes: a prospective study and meta-analysis. *Archives of Internal Medicine*.

[8] de Munter JSL, Hu FB, Spiegelman D, Franz M, van Dam RM (2007). Whole grain, bran, and germ intake and risk of type 2 diabetes: a prospective cohort study and systematic review. *PLoS Medicine*.

[9] Weickert MO, Roden M, Isken F (2011). Effects of supplemented isoenergetic diets differing in cereal fiber and protein content on insulin sensitivity in overweight humans. *American Journal of Clinical Nutrition*.

[10] Pereira MA, Jacobs DR, Pins JJ (2002). Effect of whole grains on insulin sensitivity in overweight hyperinsulinemic adults. *American Journal of Clinical Nutrition*.

[11] US Department of Agriculture (2007). The Food and Nutrition Service Handbook 901. *Food and Nutrition Service*.

[12] Centers for Disease Control and Prevention (2012). *National Health and Nutrition Examination Survey*.

[13] Hill RJ, Davies PSW (2001). The validity of self-reported energy intake as determined using the doubly labelled water technique. *British Journal of Nutrition*.

[14] Huang TTK, Roberts SB, Howarth NC, McCrory MA (2005). Effect of screening out implausible energy intake reports on relationships between diet and BMI. *Obesity Research*.

[15] Centers for Disease Control and Prevention Growth Charts. http://www.cdc.gov/growthcharts/clinical_charts.htm#Set1.

[16] Ogden CL, Carroll MD, Curtin LR, McDowell MA, Tabak CJ, Flegal KM (2006). Prevalence of overweight and obesity in the United States, 1999–2004. *Journal of the American Medical Association*.

[17] Slavin JL (2005). Dietary fiber and body weight. *Nutrition*.

[18] Johnson L, Mander AP, Jones LR, Emmett PM, Jebb SA (2008). Energy-dense, low-fiber, high-fat dietary pattern is associated with increased fatness in childhood. *American Journal of Clinical Nutrition*.

[19] Gropper SS, Acosta PB (1987). The therapeutic effect of fiber in treating obesity. *Journal of the American College of Nutrition*.

[20] Davis JN, Alexander KE, Ventura EE (2007). Associations of dietary sugar and glycemic index with adiposity and insulin dynamics in overweight Latino youth. *American Journal of Clinical Nutrition*.

[21] Cheng G, Karaolis-Danckert N, Libuda L, Bolzenius K, Remer T, Buyken AE (2009). Relation of dietary glycemic index, glycemic load, and fiber and whole-grain intakes during puberty to the concurrent development of percent body fat and body mass index. *American Journal of Epidemiology*.

[22] Ventura E, Davis J, Byrd-Williams C (2009). Reduction in risk factors for type 2 diabetes mellitus in response to a low-sugar, high-fiber dietary intervention in overweight Latino adolescents. *Archives of Pediatrics and Adolescent Medicine*.

[23] Howarth NC, Saltzman E, Roberts SB (2001). Dietary fiber and weight regulation. *Nutrition Reviews*.

[24] Slavin JL, Savarino V, Paredes-Diaz A, Fotopoulos G (2009). A review of the role of soluble fiber in health with specific reference to wheat dextrin. *Journal of International Medical Research*.

[25] Bell SJ (2011). A review of dietary fiber and health: focus on raisins. *Journal of Medicinal Food*.

[26] Heikkila HM, Schwab U, Krachler B, Mannikko R, Rauramaa R (2012). Dietary associations with prediabetic states—the DR's EXTRA Study (ISRCTN45977199). *European Journal of Clinical Nutrition*.

[27] Kim H, Stote KS, Behall KM, Spears K, Vinyard B, Conway JM (2009). Glucose and insulin responses to whole grain breakfasts varying in soluble fiber, *β*-glucan: a dose response study in obese women with increased risk for insulin resistance. *European Journal of Nutrition*.

[28] Weickert MO, Möhlig M, Schöfl C (2006). Cereal fiber improves whole-body insulin sensitivity in overweight and obese women. *Diabetes Care*.

[29] Chandalia M, Garg A, Lutjohann D, Von Bergmann K, Grundy SM, Brinkley LJ (2000). Beneficial effects of high dietary fiber intake in patients with type 2 diabetes mellitus. *The New England Journal of Medicine*.

[30] Bajorek SA, Morello CM (2010). Effects of dietary fiber and low glycemic index diet on glucose control in subjects with type 2 diabetes mellitus. *Annals of Pharmacotherapy*.

[31] Um SH, D’Alessio D, Thomas G (2006). Nutrient overload, insulin resistance, and ribosomal protein S6 kinase 1, S6K1. *Cell Metabolism*.

[32] Bailey RL, Mitchell DC, Miller C, Smiciklas-Wright H (2007). Assessing the effect of underreporting energy intake on dietary patterns and weight status. *Journal of the American Dietetic Association*.

